# Reproducibility of myocardial salvage in acute myocardial infarction by the use of Contrast-Enhanced Magnetic Resonance Imaging

**DOI:** 10.1186/1532-429X-11-S1-P153

**Published:** 2009-01-28

**Authors:** Mahdi Sareban, Hubertus Engelhardt, Ingo Eitel, Matthias Gutberlet, Gerhard Schuler, Holger Thiele

**Affiliations:** University of Leipzig – Heart Center, Leipzig, Germany

**Keywords:** Acute Myocardial Infarction, Infarct Size, Surrogate Endpoint, Microvascular Obstruction, Delay Enhancement

## Introduction

Using mortality as an endpoint in studies comparing different reperfusion strategies in STEMI requires increasingly large sample sizes to test advances with existing therapy. Thus, there is rising interest in surrogate endpoints allowing a reduction in sample size. One of those measures is myocardial salvage which can be assessed retrospectively by T2-weighted and delayed enhancement images as shown in animal studies [1]. Currently there are only limited data in humans with acquisition of T2-weighted images not covering the full ventricle [2]. In order to establish myocardial salvage MR imaging as a surrogate endpoint there is the need of convincing reproducibility data. So far reproducibility has been established only for infarct size measurements [3].

## Purpose

To assess the reproducibility of myocardial salvage measurements in a group of patients with acute myocardial infarction scanned at two subsequent days with a second contrast agent injection.

## Methods

In 20 patients with acute STEMI transverse electrocardiographically gated T2-weighted triple-inversion-recovery images were acquired to assess the area at risk and delayed enhancement (DE) images using a 3D inversion recovery gradient echo sequence assessed the infarct size. All patients underwent a repeated measurement the subsequent day starting with new scout images and using the same dose of the contrast agent. Area at risk and infarct size were determined as the percentage of left ventricular mass (%LV) by two independent observers and subsequently the myocardial salvage index was calculated as area at risk-infarct size/area at risk. In addition extent of microvascular obstruction was assessed. Inter-, intraobserver variabilities and reproducibility data were calculated according to standard definitions and compared by the method of Bland and Altman. Image quality was assessed by a score ranging from 0–4 (0 = not assessable; 4 = optimal image quality).

## Results

The mean difference between percutaneous coronary intervention and MR imaging was 2.8 ± 1.3 days. All images were suitable for analyses with a mean image quality of 3.2 ± 0.6 for DE-images and 2.5 ± 0.6 for T2-weighted-images. The area at risk was as high as 47.4 ± 11.7%LV and the infarct size 20.2 ± 9.6%LV. The corresponding myocardial salvage index was 57.7 ± 14.9 (range 23.4–75.8). In 17 (85%) patients there was evidence of microvascular obstruction. Area at risk difference (bias) between scan I and scan II was 1.2%LV and limits of agreement were ± 7.5%LV. The results for infarct size were 0.5%LV ± 2.6%LV limits of agreement. The resulting bias for myocardial salvage index was -0.3 with limits of agreement of ± 5.0. Intra- and interobserver variability was low with a mean bias of -0.7 (limits of agreement ± 4.6) and -0.0 (limits of agreement ± 4.7), respectively.

## Conclusion

The multimodal MRI approach is an excellent tool for myocardial salvage assessment with excellent reproducibility in acute STEMI. It has therefore the potential to serve as a surrogate endpoint to uncover advantages of new reperfusion strategies and allows a further reduction in the sample size of clinical trials. The previously shown excellent reproducibility for infarct size measurement could be confirmed in this trial.
